# Supplementary figures and images for: Improved Protocols of ITS1-Based Metabarcoding and Their Application in the Analysis of Plant-Containing Products
Source: Genes (Basel). 2019 Feb 7;10(2):122. doi: 10.3390/genes10020122 (PMC6409534; doi:10.3390/genes10020122)

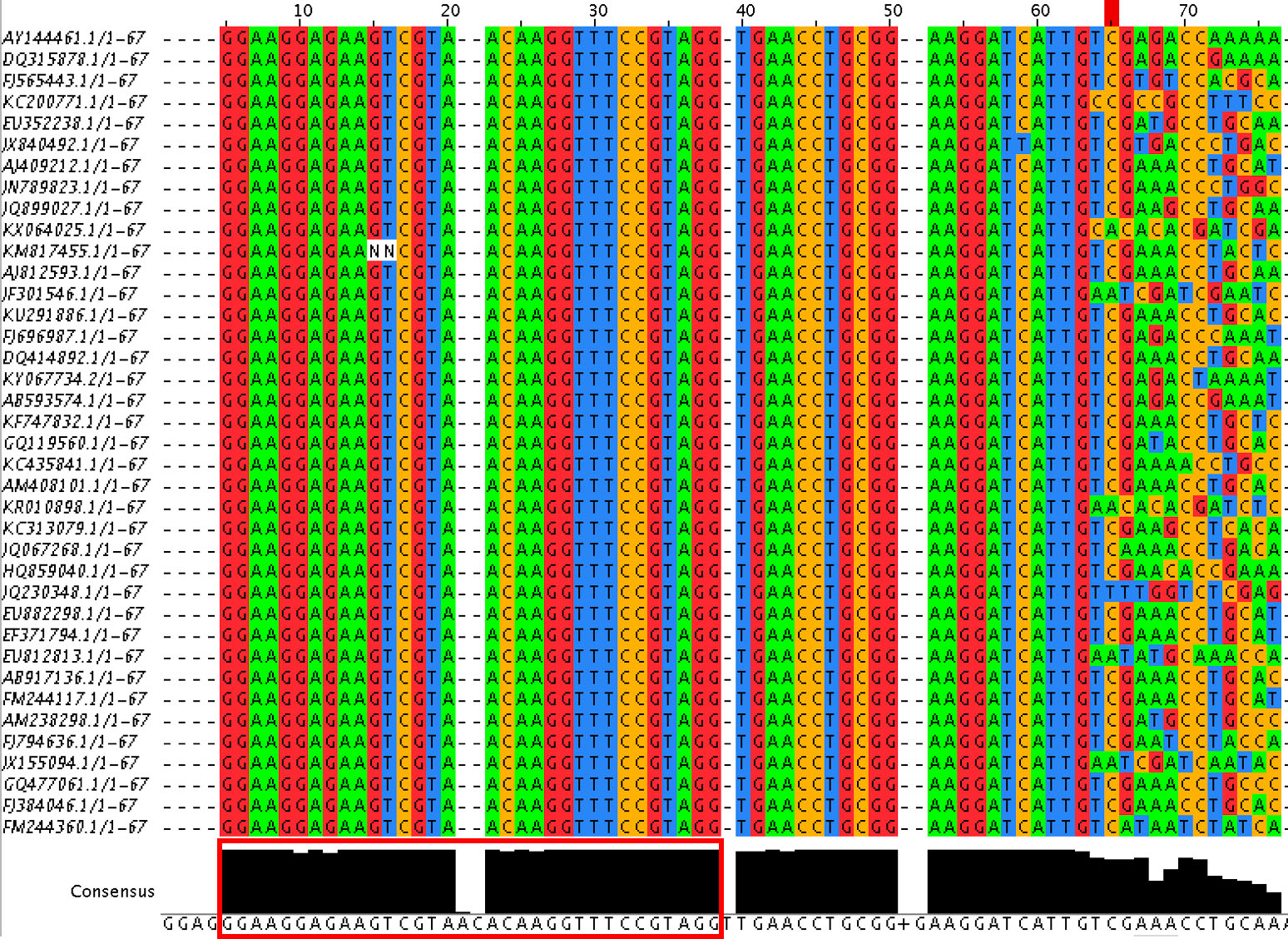

Supplement: Supplementary file 1 [file genes-10-00122-s001.zip › Suppl_fig1.jpg]

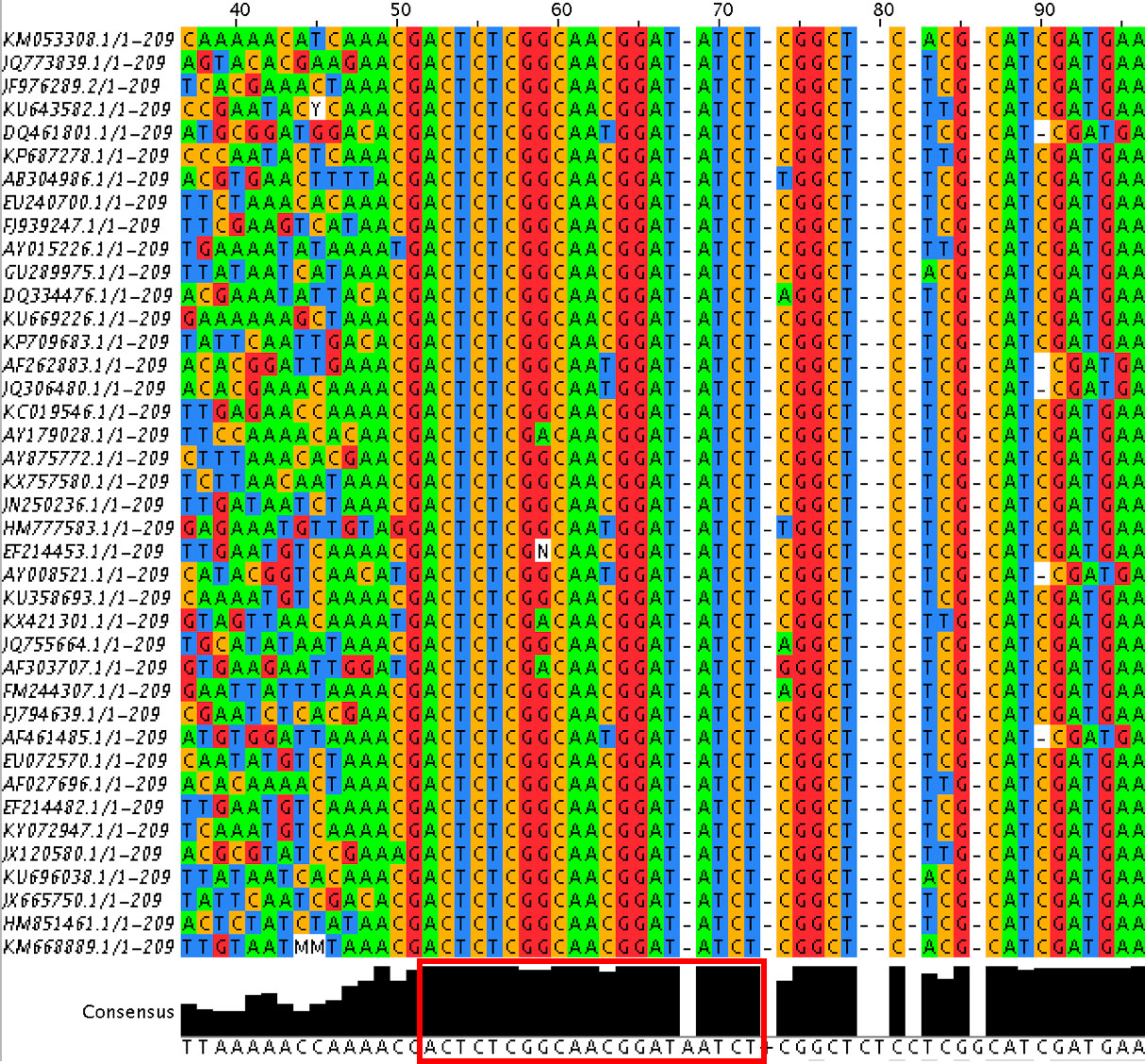

Supplement: Supplementary file 1 [file genes-10-00122-s001.zip › Suppl_fig2.jpg]

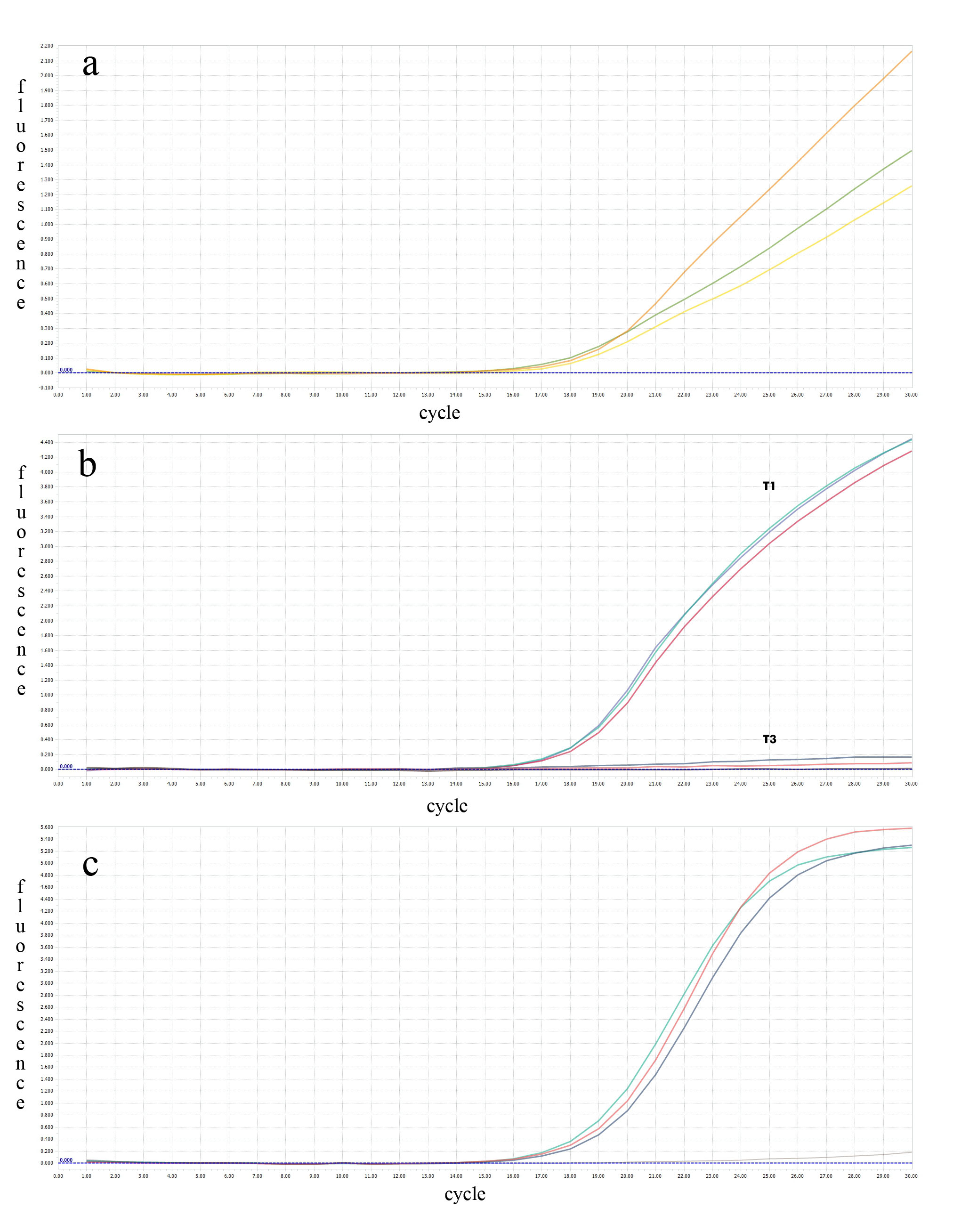

Supplement: Supplementary file 1 [file genes-10-00122-s001.zip › Suppl_FigureS3.jpg]
